# What is the role and authority of gatekeepers in cluster randomized trials in health research?

**DOI:** 10.1186/1745-6215-13-116

**Published:** 2012-07-26

**Authors:** Antonio Gallo, Charles Weijer, Angela White, Jeremy M Grimshaw, Robert Boruch, Jamie C Brehaut, Allan Donner, Martin P Eccles, Andrew D McRae, Raphael Saginur, Merrick Zwarenstein, Monica Taljaard

**Affiliations:** 1 Rotman Institute of Philosophy, University of Western Ontario, London, ON, N6A 5B8, Canada; 2 Schulich School of Medicine and Dentistry, University of Western Ontario, London, ON, N6A 5C1, Canada; 3 Department of Medicine, University of Western Ontario, 339 Windermere Road, London, ON, N6A 5A5, Canada; 4 Department of Epidemiology and Biostatistics, University of Western Ontario, London, ON, N6A 5C1, Canada; 5 Ottawa Hospital Research Institute, Clinical Epidemiology Program, Civic Campus, 1053 Carling Avenue, Ottawa, ON, K1Y 4E9, Canada; 6 Department of Medicine, Faculty of Medicine, University of Ottawa, Ottawa, ON, K1H 8L6, Canada; 7 Graduate School of Education and Statistics Department, Wharton School, University of Pennsylvania, 3700 Walnut Street, Philadelphia, PA, 19104, USA; 8 Department of Epidemiology and Community Medicine, University of Ottawa, Ottawa, ON, K1H 8M5, Canada; 9 Robarts Clinical Trials, Robarts Research Institute, London, ON, N6A 5K8, Canada; 10 Institute of Health and Society, Newcastle University, Baddiley-Clark Building, Richardson Road, Newcastle upon Tyne, NE2 4AX, UK; 11 Division of Emergency Medicine, Foothills Medical Centre, University of Calgary, 1403 29th Street NW, Calgary, AB, T2N 2T9, Canada; 12 Department of Medicine, Ottawa Hospital Research Institute, University of Ottawa and Ottawa Hospital, 1053 Carling Avenue, Ottawa, ON, K1Y 4E9, Canada; 13 Centre for Health Services Sciences, Sunnybrook Health Sciences Centre, 2075 Bayview Avenue, Toronto, ON, M4N 3M5, Canada

## Abstract

This article is part of a series of papers examining ethical issues in cluster randomized trials (CRTs) in health research. In the introductory paper in this series, we set out six areas of inquiry that must be addressed if the CRT is to be set on a firm ethical foundation. This paper addresses the sixth of the questions posed, namely, what is the role and authority of gatekeepers in CRTs in health research? ‘Gatekeepers’ are individuals or bodies that represent the interests of cluster members, clusters, or organizations. The need for gatekeepers arose in response to the difficulties in obtaining informed consent because of cluster randomization, cluster-level interventions, and cluster size. In this paper, we call for a more restrictive understanding of the role and authority of gatekeepers.

Previous papers in this series have provided solutions to the challenges posed by informed consent in CRTs without the need to invoke gatekeepers. We considered that consent to randomization is not required when cluster members are approached for consent at the earliest opportunity and before any study interventions or data-collection procedures have started. Further, when cluster-level interventions or cluster size means that obtaining informed consent is not possible, a waiver of consent may be appropriate. In this paper, we suggest that the role of gatekeepers in protecting individual interests in CRTs should be limited. Generally, gatekeepers do not have the authority to provide proxy consent for cluster members. When a municipality or other community has a legitimate political authority that is empowered to make such decisions, cluster permission may be appropriate; however, gatekeepers may usefully protect cluster interests in other ways. Cluster consultation may ensure that the CRT addresses local health needs, and is conducted in accord with local values and customs. Gatekeepers may also play an important role in protecting the interests of organizations, such as hospitals, nursing homes, general practices, and schools. In these settings, permission to access the organization relies on resource implications and adherence to institutional policies.

## Background

This article is part of a series of papers examining ethical issues in cluster randomized trials (CRTs) in health research. CRTs are increasingly used in knowledge-translation research, quality-improvement research, community-based intervention studies, public-health research, and research in developing countries. Although a small but growing literature has explored the ethical aspects of CRTs, CRTs raise difficult issues that have not been addressed adequately. In the introductory paper in this series, we set out six areas of inquiry that must be addressed if the CRT is to be set on a firm ethical foundation [1]. These include identifying research subjects, obtaining informed consent, applying clinical equipoise, performing risk-benefit analysis, protecting vulnerable populations, and understanding the role and authority of gatekeepers in CRTs. This paper addresses the sixth of the questions originally posed, namely, what is the role and authority of gatekeepers in CRTs in health research?

Gatekeepers, sometimes referred to as guardians or cluster representation mechanisms, play a prominent role in CRTs [2,3]. Gatekeepers may be called upon to protect the interests of individual study participants, clusters, or organizations that are the setting for CRTs. The use of gatekeepers stems primarily from the difficulty of obtaining informed consent from study participants [2,4]. Previous articles in this series have called for a more restrictive view of who counts as a research subject, and from whom and when informed consent must be obtained in a CRT [5,6]. This effectively diminishes the number of cases in which it is necessary to use gatekeepers.

In this article, we consider a number of steps. First, we outline the development of the use of gatekeepers in the CRT literature. Second, we document the wide variety of roles served by gatekeepers in the protection of individual, cluster, and organizational interests in CRTs. Third, we provide a detailed ethical analysis of the authority of gatekeepers to fulfill these roles legitimately. Fourth, and finally, we consider the application of our findings using three examples.

### Development of gatekeepers in CRTs

In their discussion of CRTs, Edwards and colleagues usefully distinguished between two types of CRTs: individual-cluster and cluster-cluster trials [2]. In individual-cluster trials, interventions are administered to research subjects on an individual basis, although subjects are assigned to each arm of the trial as part of a cluster. For example, research subjects may receive a novel medical or surgical treatment. Cluster-cluster trials, by contrast, involve interventions directed at groups of study participants, such as public educational campaigns, treatment of water supplies, and pesticide use. Both individual-cluster and cluster-cluster trials may involve difficulties in obtaining informed consent, but there are greater difficulties associated with cluster-cluster trials.

As Edwards and colleagues suggest, ‘if the people within clusters are given treatments, they can in theory consent individually to the treatment(s) offered within their cluster’ [2]. In individual-cluster trials, research subjects must interact directly with researchers on an individual basis at some point, in order for the treatments to be administered. This implies that, in general, it will also be possible to interact directly with the research subject for the purposes of obtaining informed consent. Nonetheless, aspects of individual-cluster trials may interfere with the ability of researchers to obtain informed consent for all aspects of the CRT [7]. Clusters are often randomized to an arm of the study before it is possible to approach the cluster members for informed consent. In these cases, even when it is possible to obtain informed consent for other aspects of the study, obtaining consent for randomization is not possible. Further, particularly when study interventions seek to produce a change in behavior, researchers may seek to withhold details of interventions in other trial arms from the informed-consent process, in order to mitigate the risk of biasing the study outcome.

In cluster-cluster trials, the use of cluster-level interventions and cluster size may hamper the ability of the researcher to obtain the informed consent of study participants with respect to both randomization and study interventions. When clusters are large, perhaps encompassing entire communities, it may be impossible for researchers to obtain the consent of every individual cluster member. In addition, when the study intervention targets the entire cluster, it may be difficult for cluster members to avoid the intervention. As a result, individuals may be unable to meaningfully refuse participation in the trial, so long as they remain a member of the cluster in the CRT [2,3].

In response to these difficulties, Edwards and colleagues introduced the idea of gatekeepers, suggesting that ‘the decision about whether a particular cluster participates in the trial is taken by an agent who has the power to ‘deliver’ that cluster’ [2]. The role of the gatekeeper differs depending on the particular features of the CRT. In individual-cluster trials, the gatekeeper may provide permission to randomize the cluster, while the research subjects provide their consent for the study interventions and data-collection procedures [2]. In cluster-cluster trials, the role of the gatekeeper may be more expansive. In these cases, according to Edwards and colleagues, the gatekeeper ‘must consent to or decline both trial entry and the intervention as a single package’ [2]. In effect, the gatekeeper provides proxy consent for cluster members, both with respect to randomization and study interventions.

Others share the view that gatekeepers may make decisions about participation in a trial on behalf of cluster members. For instance, Donner and Klar mention community leaders and elected or appointed officials as possible gatekeepers who may provide permission to randomize or consent to study interventions on behalf of cluster members [8]. In their words, ‘it may be permissible in some studies that the decision regarding random assignment and implementation of an intervention comes from community leaders or decision-makers’ [8]. Hutton also defines gatekeepers as ‘people in either political or administrative positions who are able to give consent for those within a cluster to be randomized’ [4].

Hutton suggests that it would be desirable for a gatekeeper to agree to a set of duties for a cluster before acting as its representative. However, the gatekeeper may choose not to adopt the role of advocate and, consequently, may prevent the cluster from entering into an otherwise advantageous research partnership [4]. According to Edwards and colleagues, gatekeepers should act as an advocate for the cluster, to advance its interests and preserve its trust [2]. Thus, whether the gatekeeper should give permission to enroll the cluster in a particular trial will depend on the particular features of the CRT, and whether participation is, on balance, in the interests of the cluster. Additionally, according to Hutton, gatekeepers may play a role in informing researchers about special considerations in the cluster and in informing cluster members about the research project [4].

The views of Edwards and colleagues are also reflected in the document published by the United Kingdom Medical Research Council (MRC) entitled *Cluster Randomized Trials: Methodological and Ethical Considerations* (hereafter referred to as the ‘MRC Guidelines’). As the MRC Guidelines explain, ‘the ethical principle here is that the [gatekeeper] must act in good faith, and in this regard only in the interests of the cluster represented’ [3]. Gatekeepers may give permission to enroll subjects on behalf of a cluster, and are to do so solely on the basis of what would be in the interests of the cluster. Gatekeepers are not to enroll the cluster if the study is contrary to its interests, and they are to remain informed advocates for the cluster throughout the trial. If the gatekeeper does enroll the cluster, then the cluster should be withdrawn only if the study no longer serves its interests [3]. The MRC Guidelines also state that gatekeepers must avoid any conflicts of interest, and disclose any unavoidable conflicts [3].

The MRC Guidelines propose several safeguards to ensure that gatekeepers make their decision about whether to enroll the cluster in a way that is analogous to individual consent. For instance, the MRC Guidelines require documentation as evidence that the gatekeeper understands the interests and values of the cluster. This would show that the gatekeeper is able to make decisions about enrolment based on what cluster members would endorse if they had the opportunity to decide for themselves [3]. Ultimately, however, the MRC Guidelines acknowledge that the gatekeeper’s permission to enter the cluster into the study is not ‘truly equivalent to [individual] consent’ [3]. However, a gatekeeper’s agreement may be the best that can be done to protect individuals’ interests, given the constraints imposed by study design.

More recently, the role of gatekeepers as proxy decision-makers has been discussed in the document by the Council for International Organizations of Medical Sciences (CIOMS) entitled *International Ethical Guidelines for Epidemiological Studies* (the ‘CIOMS Guidelines’), which outlines a mechanism for protecting individual interests through consultation between researchers and community representatives. In the words of the CIOMS Guidelines, interactions between researchers and representatives should be ‘aimed at obtaining the views of people who are in effect proxies for the potential subjects’ [9]. Thus, the guidelines seem to allow gatekeepers to act as proxy decision-makers for cluster members.

### Variety of roles undertaken by gatekeepers

What roles do gatekeepers undertake in CRTs? Using a validated electronic search strategy implemented in MEDLINE [10], our research team identified a random sample of 300 published CRTs. Two reviewers abstracted detailed information about procedures in the published trial, including the identification of a gatekeeper and informed consent [11]. Of the 300 CRTs, only 69 (23 %) clearly identified a gatekeeper (although in a subsequent survey carried out among the corresponding authors of these trials, 95 % of 181 respondents indicated that the trial involved a gatekeeper). In Table 1, we present a convenience sample of 27 of these trials purposely selected by three of us (AG, CW, and MT) to document the variety of roles undertaken by gatekeepers (Table 1) [12-38]. Gatekeepers have been employed in CRTs involving athletic organizations, communities, health centers, nursing homes, schools, and workplaces. Within each type of setting, different people have served as gatekeepers. For instance, in community CRTs, government authorities, community leaders, community advisory boards, medical leaders, and index members have all acted as gatekeepers. In school-based CRTs, the gatekeeper role has been filled by local governments, school districts, and principals. The specific roles undertaken by gatekeepers have also been diverse. Below we outline eight gatekeeper roles described in the study publications.

**Table 1:** Gatekeepers and gatekeeper roles in diverse CRTs in health research

**Setting**	**Country**	**Cluster type**	**Level of intervention**	**Individual consent reported**	**Gatekeeper**	**Gatekeeper role**	**Ref.**
Athletic organizations	Norway	Sports clubs	Cluster	No	Coaches	Agreement to participate^a^	[12]
	Netherlands	Geographic regions	Individual	No	Coaches	Consent^b^	[13]
	Canada	Athletic teams	Individual	Yes	Head athletic therapist, trainer or sports medicine physician	Identification of cluster members	[14]
Communities	USA	Cities	Cluster	No	Community medical leaders	Permission to randomize	[15]
	USA	Rural communities	Cluster	Yes	Community advisory board	Cluster consultation	[16]
	Bulgaria	Social circles	Individual	Yes	Index member^c^	Identification of cluster members	[17]
	Gambia	Geographic areas/districts	Individual	Yes	Community leaders	Cluster permission	[18]
	Tanzania	Residential areas (Bazoli)	Cluster	Yes	Tanzania Institute for Medical Research	Protocol approval	[19]
	India	Villages	Individual	Yes	Local health system and community leaders	Cluster consultation; agreement to participate^a^	[20]
	Uganda	Villages	Individual	Yes	Local government authorities and local leaders	Protocol approval	[21]
Health centers	USA	Health centers	Individual	Yes	Practicing internists	Identification of cluster members; permission to approach cluster members	[22]
	UK	Primary-care practices	Cluster/ Individual	Yes	Primary-care trust administrative authority	Protocol approval	[23]
	UK	Primary-care practice locations	Cluster	Yes	General practitioners	Identification of cluster members; permission to approach cluster members	[24]
	UK	Midwives	Individual	Yes	NHS trusts^d^	Agreement to participate^a^	[25]
Nursing homes	Canada	Nursing homes	Individual	Yes	Management	Not applicable^e^	[26]
	Australia	Nursing homes	Individual	Yes	Director of nursing	Not applicable^f^	[27]
	UK	Nursing homes	Individual	Yes	Management	Agreement to participate^a^	[28]
	New Zealand	Wards within nursing homes	Individual	Yes	Senior management	Consent^b^	[29]
Schools	Germany	Classrooms	Cluster	Yes	Responsible local governments	Agreement to participate^a^	[30]
	Belgium	Classrooms	Cluster	No	Principals	Organizational permission	[31]
	USA	Classrooms	Cluster	No	School district^g^	Protocol approval	[32]
	UK	Teachers	Individual	Yes	Principals	Organizational permission	[33]
	Canada	Schools	Cluster	No	District principals	Proxy consent for cluster members	[34]
Work sites	Sweden	Work sites	Individual	No	Managers and human resources	Identification of cluster members; permission to randomize	[35]
	USA	Work sites	Cluster	No	Employee advisory board	Cluster consultation	[36]
	USA	Fire stations	Individual	Yes	Department chiefs/ union representatives	Identification of cluster members; organizational permission	[37]
	USA	Pools	Individual	Yes	Pool managers, community advisors, recreation leaders, pool directors	Cluster consultation	[38]

^a^Several studies report ‘agreement to participate’ or ‘permission to conduct the study’ although it remains unclear if these are cluster permission, protocol approval, or another gatekeeper role.

^b^Studies that report ‘consent’ from a potential gatekeeper have not specified if this is cluster permission or proxy consent for cluster members.

^c^Index members were defined the ‘leaders of Roma (gypsy) men’s social networks’.

^d^Midwives may also have acted as gatekeepers as ‘all participating midwives were given detailed training on the procedure to identify, recruit, and obtain written informed consent from participants’.

^e^Gatekeeper involvement was indicated by the statement that ‘[n]ursing homes withdrew…based on a decision by the nursing home management’, although a specific role was not given.

^f^Researchers reported that directors of nursing ‘were given the opportunity to participate in the study’ without further clarification.

^g^This study also used a joint staff service committee (principal, vice principal, and faculty) that was informed and provided support for the study. The committee was not identified as a gatekeeper.

#### *Gatekeeper roles relevant to the protection of individual interests*

Gatekeepers have undertaken a number of roles that may be understood as primarily protecting individual interests. In the context of health research, both the autonomy and welfare interests of prospective study participants may be involved. Autonomy interests include a right to decide freely and on the basis of adequate information whether to participate in a study. Welfare interests include the receipt of appropriate medical care for an illness and protection from undue research risks. Gatekeepers have given permission for randomization, provided proxy consent for cluster members, given permission to approach cluster members, and identified cluster members for researchers.

##### *Permission to randomize*

In trials that randomly assign clusters to their respective arms before cluster members can be identified, gatekeepers have been asked for permission to assign the cluster randomly to one of the study arms. For example, the Rapid Early Action for Coronary Treatment study investigated the effect of community interventions on patient responses to symptoms of myocardial infarction [15]. Researchers approached community medical leaders before randomization, and reported that ‘[a]ll communities accepted their randomized assignments’ [15].

##### *Proxy consent for cluster members*

In trials employing a cluster-level intervention for which it is not practical to obtain individual consent, gatekeepers have provided proxy informed consent on behalf of study subjects. For example, the AS! BC (Action Schools! British Columbia) study evaluated the effectiveness of a physical activity program in reducing cardiovascular risk factors in elementary school children [34]. The school principals provided permission for the participation of the school, while the teachers of grades 4 and 5 provided consent to receive the study intervention on behalf of students ‘regardless of whether parents provided consent for their children to be evaluated’ [34]. Data were not collected on children whose parents declined study participation on their behalf.

##### *Permission to approach cluster members*

Gatekeepers have also played a role in determining which cluster members researchers may approach. These gatekeepers, including human resources personnel in work-site settings and physicians in health-center studies, are in positions of responsibility to ensure the well-being and privacy of prospective study subjects. For example, in a CRT studying prevention strategies in health centers, practicing internists ‘were approached for permission to recruit from among their patient pools’ [22]. Patients were identified through a central appointment system, and eligibility was determined on the basis of geographic location.

##### *Identification of cluster members*

In cases involving clusters whose members are not easily identifiable, gatekeepers have also been used to identify potential research subjects. This role differs from the above role (permission to approach cluster members) in that the gatekeeper is not in a position of responsibility with regard to cluster members; rather, the gatekeeper is merely a cluster member who is able to identify other group members. For example, in a study of HIV prevention among Roma men, researchers identified an index member and asked him to identify members of his social group, who were then approached for study participation [17].

#### *Gatekeeper roles relevant to the protection of cluster interests*

Gatekeepers have undertaken a number of roles that may be understood as primarily protecting the interests of the cluster. Cluster interests in health research are both more complex and less well understood than the interests of individual study participants. The social groups included in CRTs are heterogeneous, ranging from sports teams to cohesive communities, and, as a result, the interests at stake may be diverse and potentially conflict. Further, whereas individual autonomy and welfare interests are well understood, the morally relevant interests of social groups have resisted definitive characterization. Broadly speaking, cluster interests at stake in health research include the identity of the group and its social structures. Identity interests include the group reputation, its values and beliefs, and its social practices and traditions. Social structures include mechanisms for deliberation and decision-making, communication, shared economy, and the provision of social services. Gatekeepers have provided consent on behalf of the cluster, been involved in cluster consultation, and provided protocol approval.

##### *Cluster permission*

In this role, an authority within a cluster determines whether to provide permission to participate in the trial on the basis of cluster interests [39]. Cluster permission is commonly provided by community leaders, including mayors or other government officials, who are presumed to have the authority to agree to study participation on behalf of the cluster. Cluster permission is independent of but is a precondition for informed consent from individual study participants. Thus, refusal of cluster permission precludes participation of the cluster in the study, whereas cluster permission means that individual cluster members may be approached for their informed consent to study participation. For example, a CRT investigating a breastfeeding education program in India approached local health-system authorities and community leaders to obtain permission to conduct the study [20]. Informed consent was obtained from research subjects only after permission from the cluster was secured.

##### *Cluster consultation*

Although cluster consultation and cluster permission may occur in the same study, they are conceptually distinct. Cluster consultation involves seeking feedback and advice on how a study should be designed and conducted, but it does not involve permission to conduct the study. Cluster consultation may involve discussion between researchers and cluster representatives in order to solicit input into all stages of the research process from study design to publication [39]. In some cases, cluster consultation has been effected through the use of a community advisory board [40]. For example, in a CRT examining a complex intervention for cancer prevention among Hispanic Americans, members from communities assigned to the intervention arm were recruited to form a community advisory board [16]. An important role of the board was to provide ‘insights as to the cultural appropriateness of different intervention activities targeted [at] both Hispanics and non-Hispanic Whites’ [16]. The community advisory board did not provide permission on behalf of the clusters.

##### *Protocol approval*

Protocol approval is normally the purview of research ethics committees, but it has also been undertaken by cluster representatives to ensure that the study is consistent with the values and priorities of the cluster. For example, researchers investigating interpersonal psychotherapy for depression in rural Uganda invited local government authorities to review the research protocol after it had been approved by a research ethics committee in the sponsor country [21].

#### *Gatekeeper roles relevant to the protection of organizational interests*

Gatekeepers have also undertaken roles in the protection of the interests of organizations involved in CRTs by providing permission on behalf of the organization. In CRTs involving organizations, such as hospitals, nursing homes, and schools, the organization may be identical to the cluster and, as a result, cluster interests and organizational interests may overlap. Nonetheless, health research may have special implications for organizations, including its effects on staff, financial costs, and legal liabilities, which make it useful to consider the organizational interests separately.

##### *Organizational permission*

Gatekeepers have provided permission to conduct a CRT on behalf of organizations that are the setting for the study, including schools and work sites (Table 1). In providing such permission, the gatekeeper should consider the effect of the study on the organization, including availability of staff, financial implications of participation, and the likelihood that members will be willing to participate. For example, in a school-based CRT evaluating a computerized intervention on dietary fat intake in adolescents, researchers approached principals for permission to include their school in the study [31]. Some principals declined to participate based on organizational interests; for example,, one principal cited ‘lack of time’ as the reason for declining to participate. [31].

### Gatekeeper authority to undertake these roles

As outlined in the previous section, gatekeepers in CRTs have fulfilled a variety of roles involving the protection of individual, cluster, and organizational interests. Unexpectedly, perhaps, the question of whether gatekeepers possess the legitimate authority to fulfill these roles remains unexamined. We believe that answers to this question are essential, because gatekeepers make decisions that have consequences for others. Gatekeeper proxy consent for cluster members may allow a study to proceed, thereby exposing people to research risks without their individual informed consent or in other cases, gatekeeper refusal to provide cluster or organizational permission may bar access to a potentially beneficial study intervention. Below we undertake an ethical analysis of the authority of gatekeepers to play the roles described in the previous section.

#### *Gatekeeper authority to protect individual interests*

Previous work by our group has significantly restricted the need for gatekeeper proxy consent for cluster members and permission to randomize cluster members. First, gatekeeper proxy consent for cluster members is not required when cluster members are not human research subjects. In our paper in this series addressing the identification of research subjects, we present a novel definition of a human research subject as ‘an individual whose interests may be compromised as a result of interventions in a research study’ [5]. We suggest that when people are affected only indirectly by cluster-level interventions (and they are not otherwise subject to intervention or interacted with, and their private health information is not collected), they are not human research subjects, and thus their informed consent is not required. For instance, knowledge-translation studies commonly intervene in health-professionals practices to align practitioner behavior with evidence-based treatment guidelines. In these CRTs, patients may be affected only indirectly by the study intervention and, insofar as the health professional’s practice is brought into line with evidence-based standards, there is no risk of it affecting their interests adversely. When patients are merely affected indirectly by the study intervention, they are not human research subjects, and neither individual informed consent nor the proxy consent of a gatekeeper is required.

Second, gatekeeper proxy consent on behalf of cluster members is not required when a research ethics committee approves a waiver of consent. In our paper on informed consent, we considered circumstances in which it may be difficult to obtain the informed consent of cluster members because of cluster-level interventions or large cluster size [6]. We suggest that if the research would not otherwise be feasible and the study involvement poses no more than minimal risk to the individual cluster members, a research ethics committee may reasonably approve a waiver of consent, meaning that individual informed consent is not required. When the requirement of informed consent has been waived, it is unnecessary for a gatekeeper to provide proxy consent for cluster members.

Third, gatekeeper permission to randomize clusters is not required so long as cluster members are approached for consent as soon as possible and before any study interventions take place. In our paper on informed consent, we pointed out that the purpose of informed consent is to allow research subjects to adopt the ends of the study as their own, thereby (partially) justifying exposing subjects to risk for the benefit of others [6]. In individual-cluster trials in which clusters are randomized before the individual participants can be approached for their informed consent, it has commonly been assumed that gatekeeper permission for randomization is required. We suggest that, on the contrary, so long as cluster members are approached for informed consent as soon as possible, and before study or data-collection interventions have taken place, the moral purpose of informed consent may be fulfilled. Under these conditions, cluster members have the opportunity to adopt the ends of the study as their own and, crucially, they may decline study participation before they are exposed to any risk for the benefit of others. Thus, in these cases, gatekeeper permission to randomize clusters is not required.

The question remains whether gatekeepers may legitimately provide proxy consent for cluster members in studies that do not qualify for a waiver of consent or in which individuals are not approached for consent as soon as possible after randomization of the cluster and before study or data-collection interventions have begun. Generally, in the ethics of research, a proxy decision-maker is called upon to provide informed consent on behalf of someone else when the prospective research subject is incapable, that is, when the subject lacks the requisite cognitive capacities to provide consent themselves [41]. In such instances, the next of kin usually serve as the proxy decision-maker, on the grounds that the next of kin are likely to know the prospective subject well and be naturally motivated to promote the subject’s welfare. A proxy decision-maker is responsible for making decisions that are in accordance with the subject’s previously expressed wishes and values, or that are consistent with the individual’s best interests [41]. Further, proxy decision-makers must strive to avoid any conflict of interest.

The circumstances in which gatekeepers might provide proxy consent in CRTs are dissimilar in important ways from the circumstances in which proxy decision-making typically occurs. In contrast to the conventional situations in which proxy consent is required, cluster members are often competent and capable of freely choosing whether a study is in accordance with their interests and values. Insofar as cluster members are competent, gatekeepers would only have the authority to provide proxy consent if the cluster members had autonomously authorized them to do so, and we take it that this is rarely the case. Further, gatekeepers typically have neither a close personal relationship with cluster members, nor a detailed knowledge of their individual wishes, values, or interests. This suggests that gatekeeper proxy consent for cluster members is doubly problematic. The provision of proxy consent on behalf of a cluster member who is competent, and who has not autonomously authorized the gatekeeper to make such decisions, violates that person’s autonomy. Further, the conditions that confer legitimacy on a proxy decision-maker, such as understanding the interests of cluster members, generally do not apply. Thus, gatekeepers are not legitimate proxy decision-makers for cluster members.

We conclude, therefore, that gatekeepers generally do not have the authority to provide proxy consent on behalf of individual cluster members. As a result, gatekeepers should not provide permission to randomize cluster members nor should they provide proxy consent for cluster members (Table 2). Studies that do not qualify for a waiver of consent or in which individuals are not approached for consent as soon as possible, and before study or data-collection interventions begin, should not proceed on the basis of proxy consent from gatekeepers.

**Table 2:** Summary of recommendations for the appropriate use of gatekeepers in cluster randomized trials (CRTs)

**Recommendation**	**Remarks**
Gatekeepers should not provide proxy consent on behalf of individuals in CRTs	The fact that cluster members are typically competent and gatekeepers do not have detailed knowledge of cluster members’ decision-making history, interests, and values undermines the legitimacy of gatekeepers as a proxy decision-makers
	Gatekeepers should not provide permission to randomize or proxy consent on behalf of cluster members, and CRTs should not proceed on the basis of such permission or proxy consent
When a fiduciary relationship exists between the gatekeeper and cluster members, as in a physician–patient or teacher–student relationship, the gatekeeper may provide permission to approach cluster member	Gatekeepers who are fiduciaries may deny permission to approach cluster members whose interests are likely to be unduly compromised by study participation
	Gatekeeper permission to approach cluster members is not appropriate where no fiduciary relationship exists between the gatekeeper and cluster members
When a CRT may substantially affect group-based interests, and a gatekeeper possesses the legitimate authority to make decisions on behalf of the cluster, gatekeeper permission to enroll the cluster in the trial should be sought	When a gatekeeper possesses legitimate authority with respect to the individuals involved and the decision at hand, the gatekeeper’s permission to enroll the cluster in the study should be sought
	Ambiguity about the authority of a gatekeeper may be reason for consultation with cluster members
	When a gatekeeper does not have the requisite authority, researchers should not approach the gatekeeper for permission to enroll the cluster in research, and a CRT ought not proceed on the basis of such permission
	Cluster permission does not supplant the need for individual informed consent from cluster members
When a CRT may substantially affect group-based interests, researchers should seek to protect these interests through cluster consultation to inform study design, conduct and reporting	Cluster consultation may be used to seek input on how the CRT ought to be conducted so as to enhance study protections and benefits for clusters
	Mechanisms may include open public forums, meetings with opinion leaders, presentations at religious or civic organizations, and the use of radio, television, or the internet
	Recommendations from cluster consultation are not binding and, where there are good reasons to do so, researchers may decline to make suggested changes to a study
When a CRT may substantially affect organizational interests, and a gatekeeper possesses the authority to make decisions on behalf of the organization, organizational permission should be sought from the gatekeeper.	Organizational interests may be separable from cluster interests in a CRT
	The gatekeeper will consider the effect on the organization, including availability of staff, financial implications of participation, and compliance with organizational policies
	Organizational permission does not supplant the need for individual informed consent from cluster members

Is there any role for gatekeepers in legitimately protecting the interests of research subjects in CRTs? We believe so. In certain circumstances, the gatekeeper may legitimately provide permission to approach research subjects, and this may protect individual interests in CRTs (Table 2). When the gatekeeper has fiduciary obligations to individual cluster members, as in a physician–patient or teacher–student relationship, the gatekeeper may be viewed as having an obligation not to allow researchers to approach a cluster member whose interests are likely to be unduly compromised. Thus, gatekeeper refusal from physicians or teachers for researchers to approach individual patients or students is an instance of the legitimate protection of individual interests.

When the relationship between gatekeeper and cluster members is not fiduciary in nature, the gatekeeper does not have the authority to be relied upon to protect the interests of the cluster members. In these situations, the gatekeeper may identify potential research subjects, but may not grant permission to approach individuals. The gatekeeper acting in this role should not be relied upon to protect the interests of the subject For instance, consider the role of the index member in the aforementioned study of HIV prevention among Roma men. Identification of cluster members may be an important role pragmatically in the conduct of the study, but the researchers and research ethics committees should be clear that it does not involve the protection of individual interests.

#### *Gatekeeper authority to protect cluster interests*

The wide variety of groups studied in CRTs presents a challenge to those trying to determine who has legitimate authority to represent and protect the interests of a cluster. CRTs may involve a variety of clusters, with varying degrees of cohesiveness; clusters may be sports teams, classrooms, nursing homes, work sites, primary-care practices, geographical areas, villages, or other communities (Table 1). Cluster interests may include the preservation of the identity of the group and the maintenance of the integrity of social structures. CRTs vary in the degree to which group-based interests may be affected by cluster participation. In some cases, such as knowledge-translation studies seeking to promote adoption by physicians of evidence-based guidelines, few group-based interests may be implicated; in other cases, implications for the cluster may be substantial. For instance, studies investigating the genetic determinants of breast cancer in Ashkenazi Jews may protect research subjects by maintaining their anonymity, yet the naming of the community in publications may lead to the perception that Ashkenazi Jews as a group are more susceptible to breast cancer and discrimination [39].

In certain cases, CRT participation may substantially affect group-based interests yet protecting these interests may be difficult. Clusters often do not have organized structures or legitimate authorities capable of speaking on their behalf. Further, where organized structures for representative decision-making are present, these structures may not have been established with the intention of making decisions about research participation [2]. Finally, as Hutton points out, a representative may refuse to adopt the functions of a gatekeeper for a cluster [4]. These situations therefore raise the question, as stated by Hutton: [4] ‘to whom should responsibility for the decision to enter the cluster be passed’?

The debate in the community-based research literature over a representative’s authority to provide permission to enroll a community in a study may provide some insight into a gatekeeper’s authority with respect to clusters. As discussed in the introductory paper in our series, recognition of community-based interests and the moral status of communities led to the formulation of the principle of respect for communities [1]. According to Weijer and Emanuel, community permission is appropriate if ‘the community has a legitimate political authority, which could be a legislative assembly, mayor, or tribal council, that has the authority to make binding decisions on behalf of its members’ [39]. The principle of respect for communities relies on identification of community or group characteristics to help determine when community consent is required and when consultation alone is appropriate. Importantly, community permission is not a substitute for individual informed consent.

When a CRT involves a well-defined community, and when the CRT may substantially affect group-based interests, the protections required by the principle of respect for communities may be applied directly. In such cases, researchers may be required to obtain permission from gatekeepers to enroll the cluster, prior to seeking individual informed consent from cluster members (Table 2). A gatekeeper may give permission for the cluster to participate in the study if they have legitimate authority with respect to the individuals involved and if their authority extends to the decision at hand. Whether the gatekeeper has legitimate political authority depends on whether, among the individuals who are significantly affected by the gatekeeper’s decisions, there is widespread satisfaction with the gatekeeper’s ability to make such decisions.

A variety of mechanisms may be used to determine whether the gatekeeper’s authority is legitimate. For instance, researchers may consult with the leaders and people in the group to understand the social dynamics of the group, and members’ satisfaction with the gatekeeper’s role. Consider the example of a community-based CRT investigating a breastfeeding education program in India, which sought consent from community leaders and health-system authorities to include their communities in the study [20]. Whether those who acted as gatekeepers in this trial had the authority to provide cluster consent depends on two conditions: whether the members of the community understood the gatekeepers’ roles as including the authority to make these decisions, and whether they were largely satisfied with the institutions involved, that is, the political system used to select community leaders and the local health system. Individual satisfaction with these institutions will be based not only on past decisions that have been made by the institutions involved, but also on the decisions at hand.

If it is unclear whether a gatekeeper’s authority encompasses decisions about participation in health research, researchers should determine which method of proceeding would be most likely to be be conducive to the cluster members’ satisfaction with the institutions involved. It has been reported that the views of community leaders are generally poor substitutes for the views of individual community members [42]. This suggests that ambiguity about whether a gatekeeper’s role includes making a decision about participation in a particular CRT might be a reason for consultation with cluster members. Cluster consultation is necessary because it increases the likelihood that cluster members will be satisfied with the institutions involved, and the continued legitimacy of the institutions involved depends on the satisfaction of the people whom they purport to represent.

When CRTs involve clusters other than well-defined communities, gatekeepers typically will not have legitimate authority to speak on behalf of the cluster. In such cases, researchers should not approach gatekeepers for permission to enroll the cluster in research in order to protect group-based interests, and a CRT should not proceed on the basis of such permission (Table 2).

Cluster consultation is a second mechanism for the protection of group-based interests in CRTs. As we have seen, cluster consultation may usefully augment cluster permission, particularly in cases in which there is uncertainty about the gatekeeper authority. When a CRT may substantially affect group-based interests, cluster consultation may usefully and legitimately protect these interests even in the absence of a legitimate political authority (Table 2). Thus, compared with cluster permission, we understand cluster consultation to have a broader application to CRTs as a protection for group-based interests. Cluster consultation involves a partnership between researchers and community members, from research design to publication [43]. The degree to which a cluster can participate will depend on community characteristics and cohesiveness [39]. Cluster consultation may be sought when the cluster has a common history, common culture, or other characteristics that provide cohesiveness to the group. In these cases there are several aspects of the research endeavor in which cluster members may take part, including consultation over protocol development, involvement in the conduct of research, dissemination of information, and publication of results [39].

The diversity of social groups in CRTs poses a practical challenge to effective cluster consultation. Dickert and Sugarman have usefully described the goals of consultation as enhanced protection, enhanced benefits, legitimacy, and shared responsibility [44]. Possible means of soliciting feedback on a study include open public forums, meetings with opinion leaders, presentations at religious or civic organizations, and the use of radio, television or the internet. As previously stated, cluster consultation differs from cluster permission in that researchers are seeking input on how the study should be conducted, not whether it ought to be conducted. According to Dickert and Sugarman ‘it would be disingenuous to enter into a consulting arrangement where the consulting party does not intend, *ex ante*, to take the consultants’ advice. If relevant consultants have strong negative reactions or endorse particular modifications, those reactions or modifications have significant moral force and warrant respect and careful consideration’ [44]. Although they are morally weighty, recommendations from cluster consultation are not binding, meaning that ‘investigators may sometimes justifiably act contrary to such opinions’ [44].

The gatekeeper function of protocol approval relates to the practice of cluster consultation, insofar as cluster members may participate in all stages of the research process, including providing feedback on protocol development. The partnership model requires cluster feedback to ensure the appropriateness of the study for the clusters involved. To this end, research ethics committees, whose mandate is to review study design and protocols, include community representatives among their members. Cluster approval of the study protocol may be appropriate when the research ethics committee is not representative of the community in which research is taking place.

#### *Gatekeeper authority to protect organizational interests*

Another important role of gatekeepers is the protection of organizational interests. Gatekeepers may protect or promote organizational interests by providing permission in the name of an entire organization, such as a hospital, nursing home, or school, to participate in a study (Table 2). A gatekeeper’s agreement to allow an organization to participate in a CRT may provide opportunities for individuals within that organization to participate in and benefit from research, but a gatekeeper’s refusal of permission may mean that individuals within the organization may be denied participation in potentially beneficial research. Thus, the interests of the organization may conflict with the interests of individuals within it. An organization’s administrators and managers will be guided by their legal and professional responsibilities to act in ways that promote the safety and privacy of their members, and promote the proper functioning of the organization itself.

Although it may be useful to conceive of these two sets of obligations as distinct, it should be noted that they are not entirely independent of one another. Insofar as being part of the organization serves the interests of its members, then presumably, serving the interests of the organization also goes some way towards serving the interests of its members. Gatekeepers acting on behalf of organizations may legitimately make the decision about whether the organization will participate in a CRT because they are considered to be well situated to judge organizational interests. This may simultaneously serve the interests of those within the organization. Nevertheless, although an organizational gatekeeper may decide to allow researchers to have access to individual employees, such permission is not a substitute for individual informed consent.

### Practical implications

In this section, we illustrate the practical implications of our ethical analysis (summarized in Table 2) by considering three examples involving CRTs conducted in school, community, and healthcare settings.

#### *Example 1: improving the recognition of depression in adolescence*

The recognition of depression in adolescence is crucial to providing mentally ill young people with early access to treatment. In a school-based CRT evaluating an intervention aimed at improving the ability of teachers to identify students with depression [33], the main outcome measure was the ability of teachers to identify depressed students who were independently diagnosed by psychological testing. In the study, 151 teachers in 8 schools in central Scotland were randomized to the intervention and control arms. All teachers were given class lists of students, and asked to identify students they believed to be depressed. One week later, all the teachers participated in an educational workshop on the identification of depression. On the day of the workshop, all teachers were again given class lists of students and asked to identify depressed students; teachers in the control arm completed the task before the educational session, and those in the intervention arm completed it after the session. In the same time period, 1,911 students aged 12 to 15 years were assessed for depression by a standard questionnaire for depressive symptoms. Those with high scores underwent a structured clinical interview to diagnose depression. Letters of information were sent to all parents, and they were given the opportunity to opt their child out of the CRT. Students not removed by their parents then attended an information session, and were invited to provide written, informed consent to study participation. Without any teacher training, about half of the students who were depressed were identified by at least one of their teachers, and the study intervention did not improve the identification of depressed students. This study raises a number of questions. Who are the gatekeepers in this study? Teachers? Principals? Whose interests do they protect? Do they have the authority to fulfill these roles?

Both the students and teachers participating in this CRT were human research subjects and their informed consent was required. It does not appear that clusters were randomized before students were approached for informed consent. Parents were informed of the study and given the opportunity to remove their children from it. Insofar as parents’ consent was needed, mechanisms than other opt-out forms would serve the goal of providing parents with the opportunity to refuse or consent to their child’s participation in the research. For example, the researchers could have instead used the opposite approach, requiring that parents sign and mail back the consent form if they were willing to allow their children to participate. This would have been more likely to ensure that the parents understood the aims and methods of the trial, and that they in fact agreed to their child’s participation.

However, researchers also gave students who were not removed from the study the opportunity to provide written informed consent, after attending a 45-minute information session. Thus, on balance, student interests were adequately protected by informed-consent procedures and the research ethics committee review. All of the teachers involved in the study received study and data-collection interventions. The study did not report that informed consent was obtained from teachers (an important omission). Because the study intervention was directed at teachers and not entire classrooms, there were no prominent group-based interests at stake in this study. Thus, as there was no need for cluster permission or consultation in this study, the teachers were not gatekeepers in this case.

The CRT had substantial implications for participating schools: researchers were granted access to facilities, students, and staff; students were made available for the psychological questionnaire and follow-up interviews; and teachers were given release time for the educational workshop and data collection. Thus, the school principals had an important gatekeeper function in this study in determining the ‘willingness and ability of the school to conduct the study within the research time frame and on the availability of teachers who could be released to participate in the teaching intervention’ [33]. This decision clearly falls within their remit as the senior administrator of the school. Thus, based on the study report, the gatekeeper role was a legitimate one for the principals to perform.

#### *Example 2: HIV prevention among women in low-income housing developments*

This study sought to reduce risk behaviors for HIV in women who are impoverished, belong to minority groups, and live in low-income, inner city housing developments [45]. The place-based CRT studied women in eighteen low-income housing developments in five cities in the USA, randomizing them to either an intervention group or a control group. In the intervention group, opinion leaders were identified, and these participated in a risk-reduction workshop and formed local women’s health committees. These committees organized a variety of community events to reach all women tenants. The primary outcome measure was change in reported HIV-related risk behaviors. Support and approval for the study was obtained from local Housing and Urban Development offices and the management of local housing developments. Focus groups were conducted with tenant-management organizations, healthcare providers, political leaders, and others to develop an appropriate and widely acceptable protocol. All women in the housing developments were invited to fill out anonymous questionnaires about HIV risk-related behaviors at baseline and at the end of the intervention period. The study concluded that the community-level behavioral interventions were successful at reducing HIV risk-related behaviors.

The questions raised by this study include the following. Who are the gatekeepers in this study? Opinion leaders? Local housing development officials? Whose interests do they protect? Do they have the authority to fulfill those roles?

The study evaluated the effects of a community-level behavior-change intervention strategy on reducing HIV risk-related behaviors in women in low-income housing developments. Clusters in this study may also be conceived of as involving communities, because prospective research subjects were selected on the basis of shared geographic and social characteristics. Further, CRT participation may have an effect on the identity interests of the clusters, including the possibility of stigmatization and discrimination resulting from perceptions of HIV risk in the group. However, no easily identifiable authority existed that could make binding decisions about participation on behalf of cluster members and on the basis of cluster interests. Therefore, cluster permission in this study was not appropriate, but cluster consultation was appropriate. The appropriate role of gatekeepers in cluster consultation includes providing feedback on protocol development, ensuring that the goals of the study are in accordance with the interests of the cluster, and ensuring that the study is sensitive to the concerns of cluster members. We view the focus groups conducted in this study, which included tenant-management organizations, healthcare providers, political leaders, and others, as motivated by the need for cluster consultation.

Organizations such as housing developments also have interests that may require gatekeeper protections. To access buildings and conduct research activities using resources from the housing developments, management may be approached as gatekeepers to provide access to institutional resources. It is important to clarify the interests that managers are to take into account in making their decisions. The role of the managers is to protect organizational interests. They are not in a position to represent or protect individual interests or cluster interests.

#### *Example 3: effects of redesigned community postnatal care on women’s health at 4 months after birth*

Despite advances in postnatal care, a substantial proportion of women experience physical and psychological disorders after childbirth. This study evaluated a new model of community postnatal care delivered by midwives in an attempt to improve physical and mental symptoms, including depression, after childbirth [46]. The unit of randomization in the study was the general (family) practice. In total, 120 practices were identified from a randomly selected list of general practitioners in the West Midlands region in the UK, and were approached to take part in the trial. Agreement was obtained from all practice partners and from midwifery managers in the local UK National Health Service, before approaching midwives within each practice for participation in the trial [46]. From 36 general practices that agreed to participate (17 randomized to the intervention group and 19 to the control group), 42 and 38 midwives, respectively, recruited women and provided care. Patients were informed of the study between 34 weeks’ gestation and the first visit, and their written informed consent was obtained. Midwives in the intervention arm were trained to implement a new model of care that was tailored to the individual needs of patients after birth and was based on 10 evidence-based guidelines that had been developed for the study, while midwives in the control arm provided usual care. All midwives used a checklist of physical and psychological symptoms, which was administered at the first visit, 10 days later, 28 days later, and at the discharge visit 10–12 weeks later. A postnatal depression scale was used to screen patients at 28 days and at the discharge visit. The study intervention significantly improved women’s mental health, but it had no effect on their physical health.

The questions raised include the following. Who are the gatekeepers in this study? General-practice partners and midwifery managers? Midwives? Whose interests do they protect? Do they have the authority to fulfill these roles?

Both patients and midwives participating in this CRT were human research subjects, and their informed consent was required. In this case, practice clusters were randomized before the patients (pregnant women) could feasibly be identified and approached for informed consent. However, patients were approached for informed consent as soon as possible after they were identified (between 34 weeks gestation and the first visit) and before any study intervention or data-collection procedures were carried out. Thus, gatekeeper permission to randomize was not required. Patient interests were adequately protected by research ethics committee review and the informed-consent procedures. The informed consent of the midwives in the study was also obtained. In this case, the midwives were not the gatekeepers. The study interventions and data-collection procedures were carried out after the patients’ consent was obtained, and there were no substantial group-level interests at stake.

The CRT did have substantial implications for the participating general practices. The midwives received training, and the researchers required access to patient records. Thus, the general-practice partners and midwifery managers had a gatekeeper role with regard to the organizational interests of the practices and the midwives working in them. Gatekeepers had a role to ensure that there were adequate resources, in terms of personnel and finances, for the general practice to participate in the study. Further, the gatekeeper had to see that researcher access to confidential patient records was conducted in accordance with organizational policies and legal requirements. These decisions fall within the remit of the general-practice partners and midwifery managers, hence they have the authority to legitimately fulfill the role of gatekeeper.

## Conclusion

The use of gatekeepers in CRTs arose from the challenges that the design features of CRTs pose for obtaining individual informed consent. However, using an appropriately restrictive definition of a research subject, determining when a waiver of consent may be allowable, and paying strict attention to those instances in which informed consent may not be required, helps allay many of these concerns and diminishes the need for gatekeepers in CRTs. We have suggested that gatekeepers may be called upon to protect the interests of individuals, clusters, and organizations, but that these roles may conflict in certain cases and, accordingly, ought to be viewed as distinct and separate. A gatekeeper may have the authority to protect the interests of one of these categories, but not necessarily any others’.

We have suggested that gatekeepers cannot legitimately provide proxy consent on behalf of cluster members. The ethical principle of respect for communities and notions of community permission and consultation provide a useful model for the protection of cluster interests. In a restrictive set of cases, a gatekeeper may legitimately protect cluster interests through the mechanism of cluster permission. It must be remembered that cluster permission does not supplant the need for informed consent from cluster members. Cluster consultation may meaningfully protect cluster interests in cases in which cluster permission does not apply. Finally, gatekeepers may control access to organizations, such as general practices, hospitals, and schools, by granting permission for investigators to conduct CRTs using their facilities, resources, and personnel.

### Note

We have created a wiki webpage to facilitate an open discussion about the ideas expressed in this and other papers published in the series on ethical issues in CRTs. Please enter your thoughts and comments at http://crtethics.wikispaces.com.

## Competing interests

JCB, AG, ADM, RS, MT, CW and AW have no competing interests to declare. RB, AD, MPE, JMG, and MZ have all submitted cluster-trial protocols to ethics committees, and had difficulty explaining to them the differences between CRTs and individual patient-randomized trials.

## Authors’ contributions

AG, CW, and AW contributed to the conception and design of the manuscript. AG, CW, and AW wrote the initial draft and led the writing of subsequent versions. All authors commented on sequential drafts and approved the final version.
